# Transitioning from Preclinical to Clinical Heart Failure with Preserved Ejection Fraction: A Mechanistic Approach

**DOI:** 10.3390/jcm9041110

**Published:** 2020-04-13

**Authors:** Antoni Bayes-Genis, Felipe Bisbal, Julio Núñez, Enrique Santas, Josep Lupón, Patrick Rossignol, Walter Paulus

**Affiliations:** 1 Heart Institute, Hospital Universitari Germans Trias i Pujol, 08916 Badalona, Spain; f.bisbalvb@gmail.com (F.B.); jlupon.germanstrias@gencat.cat (J.L.); 2 CIBERCV, Instituto de Salud Carlos III, 28029 Madrid, Spain; yulnunez@gmail.com (J.N.); ensantas@gmail.com (E.S.); 3 Cardiology Service, Hospital Clínico Universitario de Valencia, Universidad de Valencia, INCLIVA, 46010 Valencia, Spain; 4 Centre d’Investigation Clinique Plurithématique 1433 -INSERM- CHRU de Nancy, Inserm U1116 & FCRIN INI-CRCT (Cardiovascular and Renal Clinical Trialists), Université de Lorraine, F-54000 Vandoeuvre-les-Nancy, France; p.rossignol@chru-nancy.fr; 5 Amsterdam Cardiovascular Sciences, Amsterdam University Medical Centers, 1105 AZ Amsterdam, The Netherlands; wj.paulus@amsterdamumc.nl

**Keywords:** heart failure with preserved ejection fraction, inflammation, atrial failure, right ventricle, pulmonary artery, renal function

## Abstract

To better understand heart failure with preserved ejection fraction (HFpEF), we need to better characterize the transition from asymptomatic pre-HFpEF to symptomatic HFpEF. The current emphasis on left ventricular diastolic dysfunction must be redirected to microvascular inflammation and endothelial dysfunction that leads to cardiomyocyte remodeling and enhanced interstitial collagen deposition. A pre-HFpEF patient lacks signs or symptoms of heart failure (HF), has preserved left ventricular ejection fraction (LVEF) with incipient structural changes similar to HFpEF, and possesses elevated biomarkers of cardiac dysfunction. The transition from pre-HFpEF to symptomatic HFpEF also involves left atrial failure, pulmonary hypertension and right ventricular dysfunction, and renal failure. This review focuses on the non-left ventricular mechanisms in this transition, involving the atria, right heart cavities, kidneys, and ultimately the currently accepted driver—systemic inflammation. Impaired atrial function may decrease ventricular hemodynamics and significantly increase left atrial and pulmonary pressure, leading to HF symptoms, irrespective of left ventricle (LV) systolic function. Pulmonary hypertension and low right-ventricular function are associated with the incidence of HF. Interstitial fibrosis in the heart, large arteries, and kidneys is key to the pathophysiology of the cardiorenal syndrome continuum. By understanding each of these processes, we may be able to halt disease progression and eventually extend the time a patient remains in the asymptomatic pre-HFpEF stage.

## 1. Introduction

The incidence of heart failure with preserved ejection fraction (HFpEF) is growing worldwide, mainly due to the overall aging of the population and the pandemic of cardiovascular risk conditions, such as hypertension, diabetes, dyslipidemia, overweight, and physical inactivity. Over the past three decades, the syndrome of HFpEF has moved from a hidden corner of HF research to the center stage [1,2,3].

HFpEF currently accounts for >50% of all HF cases, and its prevalence, relative to heart failure with reduced ejection fraction (HFrEF), continues to increase [4,5]. This fact is critically worrisome since, despite their preserved left ventricle systolic function, HFpEF patients experience high morbidity and mortality [5]. However, little therapeutic progress has been achieved [6,7,8,9,10,11,12,13], essentially because HFpEF is not a well-defined clinical entity, and its pathophysiology remains incompletely understood [14].

The diagnosis of HFpEF rests on the presence of the signs and symptoms of HF and a normal left ventricular ejection fraction (LVEF) together with cardiac functional and structural alterations and elevated levels of natriuretic peptides [15]. For decades, clinical emphasis was placed on left ventricular diastolic dysfunction, which by itself does not establish the presence of HF and may incorrectly suggest the exclusive role of the ventricular myocardium in the pathogenesis of HFpEF [16,17]. The currently accepted hypothesis (discussed in this review paper) is that, besides cardiomyocyte-intrinsic defects, myocardial remodeling stems from microvascular inflammation and endothelial dysfunction, finally leading to enhanced interstitial collagen deposition and diastolic dysfunction [18].

To better understand the clinical syndrome of HFpEF, we need to better characterize the transition from the asymptomatic pre-HFpEF stage to HFpEF itself. In a recent editorial, we coined the term “pre-HFpEF stage” to describe an asymptomatic patient (absent any signs or symptoms of HF) with preserved LVEF, incipient structural heart abnormalities (similar to those reported for HFpEF), and elevated biomarker surrogates of cardiac dysfunction. In contrast to “true HFpEF”, the key clinical component of pre-HFpEF is the absence of HF signs and symptoms [19]. The evolution from American College of Cardiology Foundation (ACCF)/American Heart Association (AHA) Stage A [20], defined by a high risk for HF but without structural heart disease or symptoms of HF, to Stage B, defined by structural heart disease without signs or symptoms of HF [20], to pre-HFpEF and ultimately HFpEF is shown in Table 1.

**Figure 1:**
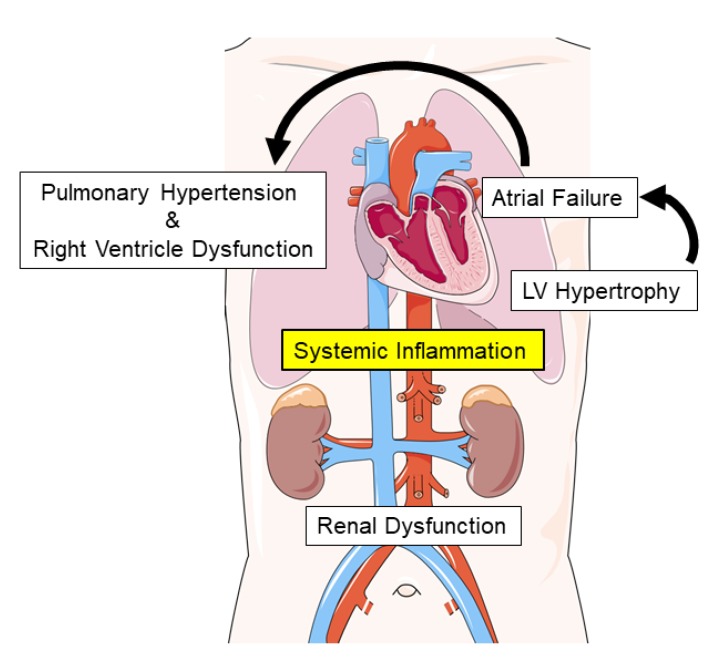
Mechanisms involved in transitioning from the pre-HFpEF stage to symptomatic HFpEF.

The present review focuses on non-left ventricular involvement in this transition with a mechanistic focus on the atria, right heart cavities, and the kidneys, and ultimately the currently accepted driver—systemic inflammation (Figure 1).

By understanding each of these processes, we may be able to halt disease progression and eventually prolong the time a patient remains in the asymptomatic and pre-HFpEF stage.

## 2. Atrial Failure

**Figure 2:**
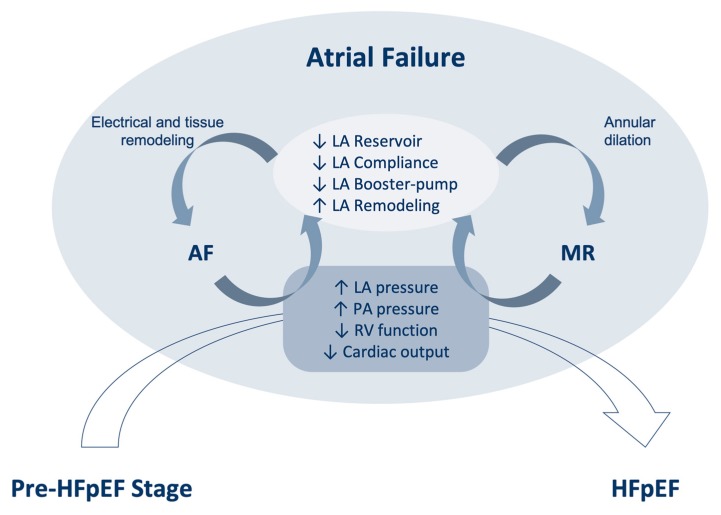
**Interaction between atrial failure and the pre-HFpEF stage.** Atrial failure is a main driver in transforming the pre-HFpEF stage into clinical HFpEF and is mainly driven by an impaired left atrial (LA) function and structure leading to increase LA and pulmonary artery (PA) pressure. The presence of atrial fibrillation (AF) and mitral regurgitation (MR) represent the rhythm and valvular manifestations of atrial failure syndrome, which further exacerbates the functional impairment of the LA and its hemodynamic consequences with adverse synergy. HFpEF, heart failure with preserved ejection fraction.

Atrial Failure is a new clinical entity defined as any atrial structural or functional abnormality (anatomical, mechanical, electrical, and/or rheological) causing impaired heart performance and symptoms and worsening the quality of life or life expectancy [21]. The impaired performance of the atrium may decrease ventricular hemodynamics and significantly increase left atrial (LA) and pulmonary pressures, leading to HF symptoms, irrespective of left ventricle (LV) systolic function (Figure 2).

Atrial involvement in HFpEF is well recognized: Impaired atrial function, increased structural remodeling, and an excess of AF incidence are consistently observed in patients with HFpEF (and to a greater extent when compared with HFrEF) [22,23,24,25]. Rather than a consequence of LV diastolic impairment, atrial dysfunction seems to be a main contributor to the onset of HF symptoms in patients in the pre-HFpEF stage. Recent evidence suggests that the LA’s function and remodeling are independently associated with (and precede) the onset of global HF in the asymptomatic healthy population [23]. A reduced atrial reserve during exercise might represent the first sign of a failing atrium in this population: compared with the controls, patients with HFpEF had a reduced atrial reservoir and conduit capacity as the only distinctive features, which was independently correlated with peak VO2 [26,27], increased exertional pulmonary pressure, and reduced cardiac output, as determined by invasive exercise pressure measurements [28]. Sanchis et al. reported that up to 45% of patients presenting with new-onset HF symptoms to a dedicated one-stop HF clinic had LA dysfunction as the unique underlying mechanism of their global HF symptoms, further supporting LA failure as an early driver of HFpEF syndrome and a key pathogenic factor [29].

Beyond its relationship to symptom onset, LA mechanical function has been consistently associated with higher adverse event rates in various cardiovascular populations [30,31]. Likewise, the impairment of specific features of LA function has been related to an increase in hard endpoints, such as hospitalization and death, in the HFpEF population [25,32].

Besides mechanical LA impairment, several atrial failure-induced conditions might further promote adverse hemodynamics and prognosis. Electrophysiological phenomena, such as intra- and inter-atrial dyssynchrony, may play a relevant role in patients with HFpEF. Late LA activation, as observed in an advanced interatrial block, leads to delayed LA contraction, shortened LA emptying, decreased LA compliance, and increased filling pressure [33]. Left atrial pacing has emerged as a promising therapy that is able to improve symptoms in this population [34]. However, the results from the randomized Left Atrial pacing in Diastolic HF (LEAD) study (NCT01618981) are needed to confirm this initial observation. Atrial fibrillation (AF) remains one of the strongest markers of worse outcomes in HFpEF. Notably, there is increasing incidence and prevalence of AF by increasing the LV ejection fraction in HF populations [24,35], which is likely driven by advanced forms of atrial myopathy in those with preserved LVEF [36]. Furthermore, both pre-HFpEF and incident atrial fibrillation have similar predisposing biomarker elevations that are indicative of microvascular endothelial activation. The presence of atrial functional mitral regurgitation is another important, often neglected, factor associated with the worsening of symptoms and a poorer outcome for HFpEF. The coexistence of atrial mitral regurgitation and HFpEF leads to a diminished capacity to increase cardiac output during exercise, likely driven by the reduced reservoir and booster functions of the LA [37] (Figure 2).

In conclusion, atrial failure is associated with the onset of HF symptoms, stroke risk, hospitalizations, and increased mortality in patients with HFpEF. Therefore, efforts to obtain a more detailed, functional characterization of their atrial structure and function are crucial to refining the mechanistic and prognostic classification of patients with pre-HFpEF. Taken together, these observations suggest that HFpEF development is not limited to LV remodeling or dysfunction but also importantly involves the LA.

## 3. Pulmonary Hypertension and Right Ventricular Dysfunction

One of the key pathophysiological phenotypes of HFpEF is the so-called “pulmonary hypertension (PH) and right ventricular dysfunction (RVD) phenotype” [38,39,40]. It is now well known that RV and PH are highly prevalent in HFpEF and are present in up to 30% and 70%–80% of patients, respectively [41]. Both parameters are key drivers of exercise intolerance and systemic congestion features [39,40], participate in the progression from subclinical pre-HFpEF to clinical HFpEF, and impact the morbidity risk or adverse events [41,42].

The exact mechanisms and risk factors causing RVD and pulmonary vascular (PV) disease in HFpEF are not fully elucidated and constitute a topic of great interest and ongoing research. In the classical paradigm, patients with HFpEF are at risk of PH because of elevated left pressure transmitted back to the pulmonary circulation, resulting in pulmonary venous hypertension. Notably, left ventricular diastolic dysfunction caused by deviation of the septum due to an increase in right ventricular pressure may also cause HFpEF. A chronic right pressure overload leads to RV remodeling and dysfunction. However, this classical paradigm does not fully explain the preponderance of RVD and PH across the whole spectrum of HFpEF patients. This process seems to be triggered and modulated by neurohormonal and inflammatory responses, in which comorbidities play an important and interrelated role [39,40,42]. In this line, a cascade of cytokines (such as tumor necrosis factor-α, interleukin-1 and 6), neurohormonal pathways (such as the endothelin-1 and adrenomedullin systems), and oxidative stress can all contribute to PV hemodynamic derangements and RV remodeling [42,43]. These processes affect the pulmonary vasculature, with muscularization of the pulmonary venules, hemangiomatosis-like endothelial cell proliferation in pulmonary capillaries, and intimal hypertrophy [44]. These changes lead to impaired pulmonary gas exchange and reduced diffusion capacity, resulting in exercise intolerance and an increased risk of mortality [45,46]. Although specific gene mutations promoting RVD and PH in HFpEF are poorly understood, shared abnormalities in PH-related signaling cascades have been found to be common in different forms of PH, reflecting in a possible genetic predisposition in a subset of HFpEF patients [47,48,49,50,51,52,53].

**Figure 3:**
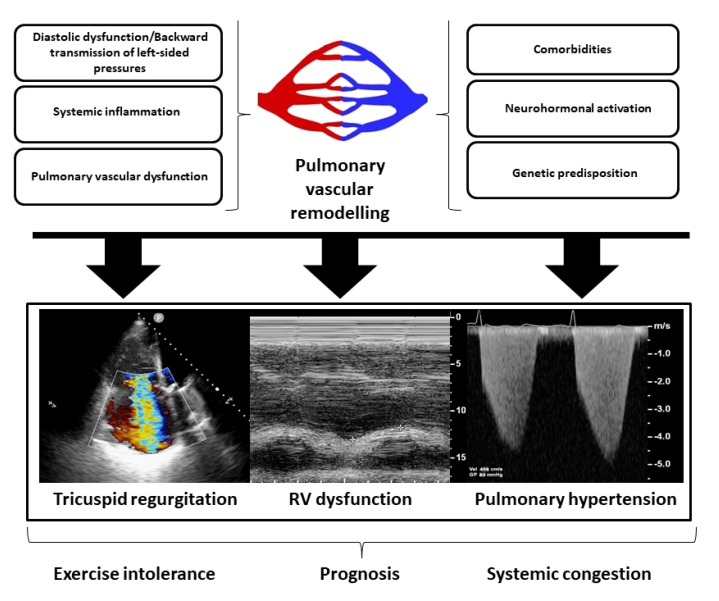
Pathophysiological pathways involved in right heart dysfunction (RHD) in heart failure with preserved ejection fraction (HFpEF). The passive backward transmission of left-sided pressure, comorbidities, systemic inflammation, neurohormonal pathways, genetic predisposition, and intrinsic lung and pulmonary vascular abnormalities are all factors interrelated in the pathogenesis of RHD in patients with HFpEF. Pulmonary vascular remodeling and the development of a pre-capillary component of pulmonary hypertension are key factors in developing RHD in HFpEF. Because of this pathophysiological pathways, right ventricular dysfunction, pulmonary hypertension, and functional tricuspid regurgitation are the main drivers of exercise intolerance, systemic congestion, and prognosis in HFpEF. RV: right ventricular.

Beyond RV function and PV disease, the competency of the tricuspid valve is crucial. Functional tricuspid regurgitation (TR) is a consequence of maladaptive RV remodeling and PH, leading to further RV enlargement and dysfunction in a vicious cycle [42]. Functional TR is a strong predictor of death and readmissions in HFpEF [54,55]. TR causes fluid retention, systemic congestion, and renal dysfunction, resulting in inflammation, neurohormonal activation, and multi-organ damage [42,56,57]. The pathophysiological pathways and important right-heart (RH) dysfunction features in HFpEF are summarized in Figure 3.

The spectrum of RVD and PH in HFpEF is very heterogeneous. Some patients do not present RH failure, whereas for others, right-sided dysfunction and PH are the dominant features [58]. Do these reflect a dynamic process or just different faces of this complex syndrome? RH failure has been classically considered to be limited to end-stage HF. Indeed, in a prospective study, 55% of patients with HFpEF died with clinical signs of RH failure [59]. However, recent data show the need to reconsider this paradigm. Borlaug et al. revealed abnormalities in pulmonary artery (PA) vasodilatation, dynamic RV-PA uncoupling, and worsening RV systolic function during exertion even in the earliest stages of the disease [60]. Thus, PH dysfunction may be present in early stages only during exercise and may be important in the transition from pre-HFpEF to overt clinical HF. The presence of abnormal RV function at rest is also a tipping point in this evolution. Low RV function and impaired RV-PA coupling are associated with incident HF in the community [61]. In another elegant study by the Mayo Clinic group, even with low values of NT-proBNP at rest, patients with abnormal baseline RV function were more likely to develop lung congestion during exercise. This involved not only increases in pulmonary capillary pressure but also increases in central venous pressure due to elevations in right atrial pressure and exercise-induced impairments in RV-PA coupling resulting in exercise-induced RVD and pulmonary congestion [62].

RV dysfunction can also progress over time. In a retrospective cohort of 271 patients with HFpEF, there was a 10% decrease in the RV fractional area change and a 21% increase in the RV diastolic area over a 4-year follow-up [63]. Interestingly, these RV changes were more pronounced than longitudinal changes on the left heart. The latter finding supports the idea that RV and LV remodeling in HFpEF is controlled by different mechanisms, namely myocardial overload for the RV and microvascular inflammation for the LV. How can we identify patients at high risk for worsening RV dysfunction? [64]. This is crucial in understanding the transition from pre-HFpEF to overt HFpEF syndrome.

Therapies targeting the PH-RVD phenotype in HFpEF have shown disappointing results, and no therapy has yet demonstrated an improvement in prognosis. Several treatments are in the pipeline, and ongoing trials will shed light on this topic in the coming years [42]. Besides RH-directed therapies, a therapeutic intervention acting on the pulmonary vasculature in the early stage of the disease is desirable. An elegant review of novel therapies targeting inflammation and cardiometabolic or extracellular abnormalities in HFpEF can be found elsewhere [39]. In addition, managing atrial fibrillation, aggressively targeting obesity, or adequately reducing congestion with optimal diuretic therapy may help slow the progression of RVD over time [63].

## 4. Renal Dysfunction

### 4.1. Epidemiological Insights

In keeping with the pre-HFpEF concept, Xhakollari et al. [65] recently reported a cross-sectional, community-based (Swedish) study within the Malmö Prevention Project aimed at investigating whether there is an early association between kidney function and echocardiographic markers of cardiac structure and diastolic function. The study population consisted of 1504 individuals with no prior history of congestive heart failure or asymptomatic left ventricular ejection fraction ≤ 40% and an estimated glomerular filtration rate (eGFR) based on cystatin C >15 mL/min/1.73 m^2^. Even mild-to-moderate renal dysfunction was found to be associated with echocardiographic indicators of diastolic dysfunction, although—notably—the associations remained significant only for men when stratifying for sex [65].

### 4.2. Pathophysiological Insights

**Figure 4:**
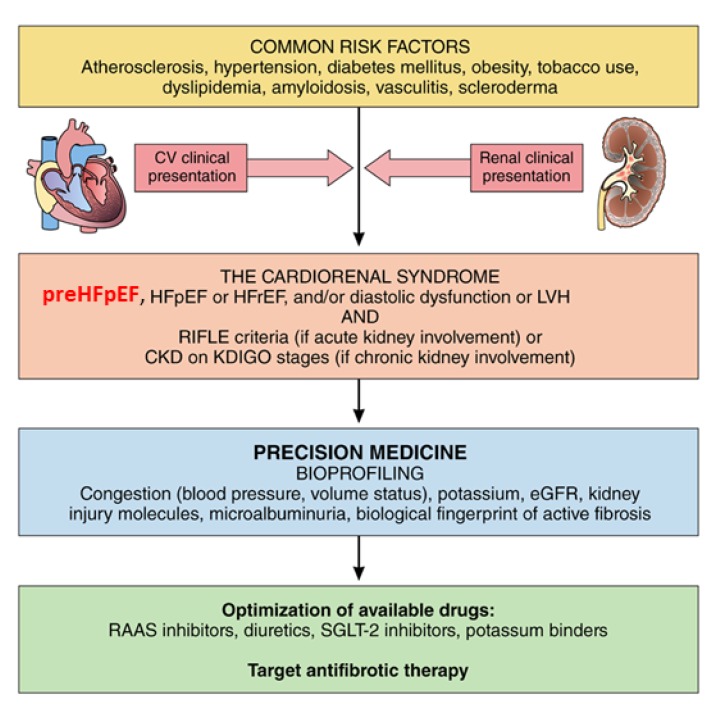
**Revisited cardiorenal syndrome** (modified with permission from Ref. 69). This figure depicts a proposed new paradigm in which common risk factors lead to cardiorenal syndrome that may have either a cardiovascular (CV) or a renal clinical presentation. Bioprofiling with clinical and biomarker information will enable precision medicine optimized to a specific profile. CKD indicates chronic kidney disease. HFpEF, heart failure with preserved ejection fraction; HFrEF, heart failure with reduced ejection fraction; KDIGO, Kidney Disease Improving Global Outcomes; LVH, left ventricular hypertrophy; and RIFLE (Risk, Injury, Failure, Loss, End-Stage).

The potential mechanisms for the development of HFpEF in chronic kidney disease (CKD) have been comprehensively reviewed [66,67]. Briefly, there is major interplay between CKD and comorbidities like hypertension, diabetes, and increased body mass index/obesity, which are key components in the development of HFpEF [68] and CKD. Recent scientific work has identified fibrosis as a common pathophysiological pathway for several categories of cardiorenal syndromes, suggesting a unifying pathogenesis [69]. In the setting of CKD, two of the most important pathophysiological features leading to HFpEF are thought to be endothelial dysfunction and chronic inflammation [66], the latter of which will be described in the following section [70]. Fibrosis, which is a common feature in heart failure and CKD, might not only be a marker but also the driver for several cardiorenal syndromes, both chronic and acute. In light of the above, we proposed a single cardiorenal syndrome umbrella with a new pragmatic and dynamic cardiorenal integrative concept (Figure 4) that combines the documentation of fibrotic pathophysiology using companion diagnostic biomarkers with therapeutic management aimed at potential common fibrotic biotargets (e.g., renin-angiotensin-aldosterone system -RAAS- inhibitors, mineralocorticoid receptor antagonists, and novel molecules) and patient-centered monitoring tools (e.g., markers of congestion and kidney function and hypertension) [69].

Regardless of the ejection fraction, heart failure symptoms are mostly related to congestion, which is one of the main predictors of poor patient outcome [68]. Hypervolemia and sodium excess are also commonly observed in CKD patients, while a subclinical volume overload is present in over 20% of patients with CKD [71]. Increased sodium can bind to the endothelial glycocalyx, causing a stiffening of endothelial cells and decreased nitric oxide (NO) levels and resulting in endothelial dysfunction. Since endothelial dysfunction and HFpEF are interrelated, this mechanism may link sodium and hypervolemia in the transition from asymptomatic pre-HFpEF to symptomatic HFpEF [66]. The role of sodium in the development of hypertension, including resistant hypertension, is mostly due to hypervolemia [72]. Evidence shows that most patients with CKD are salt-sensitive—that is, they respond to an increase in their sodium intake with a rise in blood pressure [72]. Efforts to reduce salt intake can be beneficial because of synergism with the actions of thiazides, angiotensin-converting enzyme inhibitors, and angiotensin receptor blockers, or mineralocorticoid receptor antagonists and sodium-glucose transport protein 2 (SGLT2) inhibitors, resulting in improved blood pressure control and less proteinuria, a major trigger of further kidney function alteration. Reducing blood pressure lowers the risk of new onset heart failure by as much as 40% [68]. SGLT2 inhibitors are likely to become part of the life-saving drug armamentarium in HF (following the DAPAgliflozin in patients with Heart Failure and reduced ejection fraction -DAPA-HF- trial [73], whereas HFpEF trials are still ongoing [68]), as well as in CKD [74]. Interestingly, the diuretic effect of these compounds (as assessed by increases in hematocrit) has been demonstrated and found to mediate a major component of the beneficial effect demonstrated in the EMPAgliflozin, cardiovascular OUTCOMES and mortality in type 2 diabetes -EMPA-REG OUTCOME- trial [75]. In this trial, empagliflozin reduced the risk of a broad spectrum of HF outcomes with no heterogeneity observed among patients with or without HF at baseline [76].

### 4.3. Challenges in Pre-HFpEF/HFpEF Differentiation in CKD Patients

Properly assessing congestion is of paramount importance in heart failure management [77]. However, congestion is difficult to assess, especially when symptoms are mild [77], and is even more challenging in advanced CKD patients [78]. Elevated natriuretic peptide levels, which can provide laboratory evidence of new or worsening heart failure and are one of the criteria used to define HFpEF, may result from decreased renal clearance of these markers in patients with CKD [78]. Many physical manifestations of CKD overlap with those of heart failure. Indeed, signs of volume overload and symptoms of dyspnea in patients on dialysis could be due to a missed hemodialysis session, the overestimation of dry weight, or non-adherence to dietary sodium and fluid restrictions [78]. The anemia associated with renal failure should also be considered as it may play a significant role in HFpEF development.

Dyspnea, as a key symptom in characterizing the transition from pre-HFpEF to HfpEF, may also be misleading. In a recent study by Ramalho et al. [79], the authors aimed to quantify the association of undifferentiated dyspnea with cardiac dysfunction after accounting for other potential contributors, including CKD. Their findings were based on a cross-sectional US study composed of data from 4342 Atherosclerosis Risk in Communities (ARIC) study participants 65 years and older who attended the fifth study visit (from 2011 to 2013) and had not been diagnosed with HF, chronic obstructive pulmonary disease, morbid obesity, or severe CKD (eGFR <30 mL/min/1.73 m^2^). Among the latter, 1173 (27.0%) reported dyspnea and 574 (13.2%) reported dyspnea that was moderate to severe, the latter of which was associated with left ventricular hypertrophy, as well as left ventricular diastolic and systolic dysfunction. Moderate to severe dyspnea was also associated with obstructive and restrictive findings in spirometry, renal function, anemia, lower and upper extremity weakness, depression, and obesity. After accounting for the above variables, moderate-to-severe dyspnea was found to be associated with left ventricular hypertrophy (OR, 1.30; 95% CI, 1.01–1.67; *p* = 0.04) but not with systolic or diastolic function. Among the participants with undifferentiated moderate to severe dyspnea, 58 (10.1%) actually met all the European Society of Cardiology criteria for potential HFpEF. Of note, spirometric measurements worsened across dyspnea severity categories, as did noncardiopulmonary organ function measurements, including eGFR, hemoglobin, upper and lower extremity physical function, depressive symptoms, and body mass index. The authors concluded that “contrary to our a priori hypothesis, cardiovascular measures had only a modest association with dyspnea when accounting for impairments in noncardiovascular organ systems. However, our findings highlight that many factors contribute to dyspnea in elderly people, with only a modest independent association with cardiovascular function.” Therefore, the observation that a substantial proportion of CKD patients without diagnosed heart failure may present symptoms suggestive of heart failure must be interpreted with caution [80]. Shlipak et al. used a modified Kansas City Cardiomyopathy Questionnaire (KCCQ) to assess the symptoms characteristic of heart failure (i.e., dyspnea, fatigue, and edema) among 2883 Chronic Renal Insufficiency Cohort (CRIC) US participants with moderate to severe CKD who did not report a prior diagnosis of heart failure [80]. Compared with the reference cystatin C-based estimated glomerular filtration rate of >50 mL/min/1.73 m^2^, estimated glomerular filtration rates of 40–50, 30–40, and <30 were independently associated with lower KCCQ scores with adjusted odds ratios (95% CI) of 1.38 (1.06–1.78), 1.39 (1.09–1.82), and 2.15 (1.54–3.00), respectively. More than one-fourth of the cohort had a KCCQ score below 75, a threshold value that has been used in clinical trials of established heart failure to denote at least a moderate burden of symptoms. Lower levels of eGFRcys and hemoglobin were independently associated with a higher likelihood of clinically significant symptoms, as were obesity, diabetes, and prevalent cardiovascular disease.

## 5. Systemic Inflammation

The systemic inflammation induced by comorbidities is considered to be a major player in the pathophysiology of LV dysfunction and remodeling in HFpEF [81]. Comorbidities drive LV dysfunction and remodeling through coronary microvascular inflammation, which affects both cardiomyocyte distensibility and collagen deposition in the LV myocardium. The linkage between comorbidities and biomarkers of systemic inflammation is evident from the close relationship between the number of comorbidities and the plasma high sensitivity C-reactive protein (hsCRP) level in a trial population of HFpEF patients [82]. The comorbidities accounted for in this study consisted of obesity (body mass index >30 kg/m^2^), hypertension, ischemic heart disease, atrial fibrillation, diabetes mellitus, chronic obstructive pulmonary disease, anemia, and chronic kidney disease. Especially relevant for pre-HFpEF is an early observation from the Health, Aging and Body Composition (ABC) study showing an increased hazard ratio for developing HFpEF over a 9.4-year time span when the baseline plasma tumor necrosis factor α (TNFα) level was elevated [83]. In this study, the baseline TNFα predicted the development of HFpEF but not of HFrEF [83]. Similar evidence was also derived from patients with clinical HFpEF who had higher levels of soluble interleukin 1 receptor-like 1 (IL1RL1 or ST2; *p* = 0.02), CRP (*p* = 0.01), interleukin 6 (IL6) (*p* = 0.08), and growth differentiation factor-15 (GDF-15; *p* = 0.09) than patients with HFrEF [84]. Patients with clinical HFpEF were recently extensively characterized in the BIOSTAT-CHF (A systems BIOlogy Study to TAilored Treatment in Chronic Heart Failure) program, with the determination of 92 biomarkers used for the investigation of protein–protein interactions and the evaluation of biological processes [85]. Six protein–protein interactions were unique to HFpEF, and the biological processes related to inflammation and extracellular matrix organization were overrepresented in HFpEF compared to HFrEF. The involvement of systemic inflammation in acute exacerbations of HFpEF was inferred by a study that compared inflammatory biomarkers in acute and chronic HFpEF and observed higher levels of IL6, TNFα, hsCRP, and pentraxin 3 in acute HFpEF. A proof of concept that systemic inflammation was indeed involved in exacerbations of HFpEF necessitating hospitalizations was provided by the CANakinumab, anti-inflammatory Thrombosis Outcome Study (CANTOS) trial. When canakinumab succeeded in lowering hsCRP below 2 mg/L, the likelihood of heart failure hospitalization was lower. Although this study did not discriminate between HFrEF and HFpEF, many patients were likely to suffer from HFpEF as they were old with a high prevalence of obesity, diabetes, and arterial hypertension [86]. Advanced age also predisposes one to clonal hematopoiesis of indeterminate potential (CHIP) because of mutations in Tet methylcytosine dioxygenase 2 (TET2), an epigenetic modulator. This leads to leukocytes with enhanced production of IL1 and IL6 and to a higher risk for macrovascular atherosclerotic disease and malignancies [87]. CHIP was also recently addressed in experimental pressure-overload heart failure. In this model, the TET2 mutation was mimicked via TET2 deficiency and led to increased IL-1 signaling and more extensive cardiac remodeling [88]. Because of the association of CHIP with ageing and because CHIP affects cardiac remodeling in experimental pressure overload, CHIP could contribute to the transition from pre-HFpEF to clinical HFpEF in older patients.

### From Systemic Inflammation to Diastolic LV Dysfunction

High diastolic LV stiffness is the most important hemodynamic abnormality in HFpEF, as it triggers a brisk increase in LV filling pressure during exercise and leads to incapacitating effort intolerance [89]. In a recent study, augmented diastolic LV stiffness was also evident in alleged stage B HFpEF patients [90]. Using saline infusion and lower-body negative pressure, the diastolic LV pressure–volume relation was determined over a wide range of LV filling volumes and convincingly shown to be steeper and shifted to the left, implying higher LV filling pressure at similar LV filling volumes. In this study, pre-HFpEF patients were defined by the absence of symptoms and the presence of LV hypertrophy and elevations of N-terminal pro brain natriuretic peptide (NT-proBNP > 40 pg/mL) or cardiac troponin T (cTnT >0.6 pg/mL). Evolution to clinical HFpEF was not documented in this study, which is reason for concern. In fact, a study recruiting similar patients with asymptomatic “malignant” LV hypertrophy defined by increased LV mass index on cardiac magnetic resonance imaging and elevated NT-proBNP or cTnT revealed, after 12.2 years, a larger risk for HFrEF (49.2% of incident HF) than HFpEF (40.6% of incident HF) [91]. Although HFpEF features concentric LV hypertrophy, both studies did not explicitly distinguish between eccentric and concentric LV hypertrophy. In hypertrophic cardiomyopathy, the development of eccentric remodeling is extremely rare (3.5%) [92]. One can, therefore, speculate that the evolution to HFrEF in patients with “malignant” hypertrophy was unrelated to the transition from concentric to eccentric LV remodeling but secondary to the inclusion of patients who originally already suffered from eccentric LV hypertrophy. This speculation is also supported by the high inclusion rate of black patients in the LV hypertrophy group. In the Atherosclerosis Risk in Community (ARIC) study, black and white cohorts with high prevalence of arterial hypertension (94% and 80%) showed divergent LV remodeling with a higher LV end-diastolic volume index, a higher LV mass index, and more eccentric LV remodeling in the black patient cohort [93]. This finding was ascribed to the genetically determined hypersensitivity to LV afterload among black patients. The obligatory presence of LV hypertrophy in stage B HFpEF is also questionable because in the I-PRESERVE cohort of symptomatic HFpEF patients, LV hypertrophy was present in only 29% [94].

**Figure 5:**
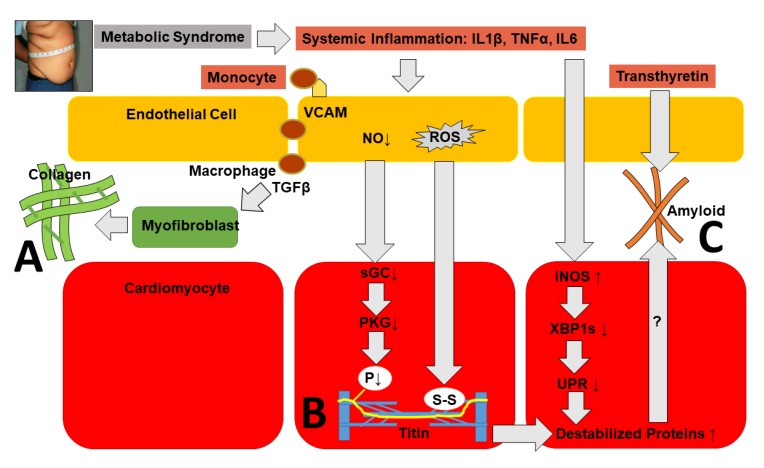
**Pathophysiological mechanisms linking systemic inflammation to diastolic LV stiffness.** (**A**): Systemic inflammation causes the endothelial expression of adhesion molecules (VCAM: vascular cell adhesion molecule). They attract monocytes that become macrophages secreting transforming growth factor β (TGF β), which stimulates myofibroblasts to deposit collagen. (**B**): Systemic inflammation lowers the endothelial production of nitric oxide (NO), soluble guanylate cyclase (sGC) activity, protein kinase G (PKG) activity, and titin phosphorylation (P). Systemic inflammation also causes the endothelial production of reactive oxygen species (ROS) with the formation of disulfide bonds (S-S) within titin. Both hypophosphorylation and S-S bonds increase titin stiffness. (**C**): Systemic inflammation boosts the expression of inducible NO synthase (iNOS). This lowers X-box binding protein 1 spliced (XBP1s) and the activation of UPR genes, which can potentially lead to the accumulation of destabilized proteins similar to transthyretin-induced amyloid deposits.

Three mechanisms have so far been proposed to link systemic inflammation to high diastolic LV stiffness in HFpEF:

(1) myocardial infiltration by leukocytes that leads to interstitial collagen deposition;

(2) altered paracrine signaling from endothelial cells that leads to stiff cardiomyocytes;

(3) deficiency of the unfolded protein response (UPR) in cardiomyocytes that leads to interstitial protein aggregation (Figure 5) [18].

In LV myocardial biopsies of HFpEF patients, the coronary microvascular endothelial expression of adhesion molecules, such as E-selectin, intercellular adhesion molecule (ICAM), and vascular cell adhesion molecule (VCAM), is upregulated. Adhesion molecules are also shed by endothelial cells into circulation where they become detectable as biomarkers. The elevation of circulating adhesion molecules in young adulthood was recently shown to predict stage B HFpEF two decades later [95]. The expression of these adhesion molecules is known to be induced by TNFα via the nuclear factor kappa-light-chain-enhancer of activated B cells (NFκB). Expression is repressed by microRNA-223, which is transported in circulation by high density lipoproteins (HDL) [96]. Metabolic syndrome, which is associated with HFpEF, features low HDL plasma levels and thereby stimulates the endothelial expression of adhesion molecules because of the low transfer of circulating microRNA-223. The endothelial expression of adhesion molecules attracts monocytes. These monocytes become macrophages secreting transforming growth factor β (TGFβ), which turns fibroblasts into myofibroblasts. These produce collagen with high tensile strength, as in scar tissue. This evidently reduces myocardial distensibility and leads to higher LV filling pressure and lower exercise tolerance [97]. In HFpEF, infiltrating macrophages are of a distinct phenotype as a result of metabolic activation that clearly differs from classical endotoxic activation [98]. Under conditions of metabolic activation, high levels of free fatty acids activate the peroxisome proliferator-activated receptor (PPR)γ, which suppresses classical NFκB signaling [99]. In HFpEF, myocardial inflammation results not only from the proinflammatory action of cytokines such as TNFα and IL6 but also from the diminished anti-inflammatory action of cytokines like IL-33. IL33 signals through suppression of tumorigenicity-2 (ST2), also called interleukin1 receptor-like 1 (IL1RL1), and exerts protective effects in the cardiovascular system. In HFpEF, myocardial periarteriolar fibrosis has recently been linked to the reduced activity of IL33 because of deficient expression of ST2 [100].

Apart from attracting monocytes, endothelial dysfunction leads to the endothelial production of reactive oxygen species (ROS) through endothelial nitric oxide (NO) synthase (eNOS) uncoupling [101] and mitochondrial dysfunction [102]. In underlying cardiomyocytes, decreased NO levels lead to lower soluble guanylyl cyclase (sGC) and protein kinase G (PKG) activity, reducing cardiomyocyte distensibility because of the lower phosphorylation of titin. ROS also directly reduces titin rigidity because of the formation within the titin molecule of disulfide bonds [103] and carbonylation [104].

Lastly, as demonstrated by Schiattarella et al. [105], elevated plasma levels of proinflammatory cytokines, such as interleukin1β (IL1β), tumor necrosis factor α (TNFα), and interleukin 6 (IL6), boost the expression of iNOS in cardiomyocytes. This ultimately leads to less activation of unfolded protein response (UPR) genes. The UPR is a regulatory system that protects the endoplasmic reticulum from an overload of improperly folded proteins. It remains to be demonstrated if the reduced activation of UPR genes effectively results in the myocardial accumulation of destabilized proteins. An argument in favor of this process is provided by the raised plasma troponin levels in HFpEF [106]. Raised plasma troponin levels are more likely to result from the myocardial accumulation of destabilized myofilamentary proteins than from cardiomyocyte cell death [107], which was never observed in the myocardial biopsies of HFpEF patients. The myocardial accumulation of destabilized proteins is observed in amyloidosis, and the mechanism proposed by Schiattarella et al. [105] links classical HFpEF to amyloidosis-related restrictive cardiomyopathy.

In conclusion, in the transition from the pre-HFpEF stage to overt HFpEF syndrome, we need to pay special attention to atrial failure, pulmonary hypertension, RVD, renal failure, and systemic inflammation above and beyond left ventricular hypertrophy. Specific targeting of each of these pathogenic mechanisms may halt disease progression and promote patient health and lifespan. The future perspectives within HFpEF management are exciting and immense; however, we will not succeed by stopping one wave at a time—we have to calm the ocean.

## Figures and Tables

**Table 1:** Transitioning from Pre- heart failure with preserved ejection fraction (HFpEF) to HFpEF phenotypes.

	AHA Stage A	AHA Stage B	Pre-HFpEF Stage	HFpEF Syndrome
**CV Risk Factors**	**+**	**+**	**+**	**+**
**LVEF ≥ 50%**	**+**	**+**	**+**	**+**
**Cardiac structural abnormalities**	**-**	**+**	**+**	**+**
**Biomarkers** ^**a**^	**-**	**-**	**+**	**+**
**Signs and symptoms of HF**	**-**	**-**	**-**	**+**

^a^ Currently natriuretic peptides; other biomarkers to be considered. AHA, American Heart Association; HF, heart failure; HFpEF, heart failure with preserved ejection fraction; LVEF, left ventricular ejection fraction.
